# Analysis of over 1 million race records shows runners from East African countries as the fastest in 50-km ultra-marathons

**DOI:** 10.1038/s41598-024-58571-0

**Published:** 2024-04-05

**Authors:** Katja Weiss, David Valero, Elias Villiger, Mabliny Thuany, Pedro Forte, Robert Gajda, Volker Scheer, Sreten Sreckovic, Ivan Cuk, Pantelis T. Nikolaidis, Marilia Santos Andrade, Beat Knechtle

**Affiliations:** 1 Institute of Primary Care, University of Zurich, Zurich, Switzerland; 2 Ultra Sports Science Foundation, Pierre-Benite, France; 3 Klinik für Allgemeine Innere Medizin, Kantonsspital St. Gallen, St. Gallen, Switzerland; 4 Faculty of Sports, University of Porto, Porto, Portugal; 5 CI-ISCE, Higher Institute of Educational Sciences of the Douro, Penafiel, Portugal; 6 LiveWell—Research Centre for Active Living and Wellbeing, Polytechnic Institute of Bragança, Bragança, Portugal; 7 Center for Sports Cardiology at the Gajda-Med Medical Center in Pułtusk, Pułtusk, Poland; 8 Department of Kinesiology and Health Prevention, Jan Dlugosz University, Czestochowa, Poland; 9 College of Sports and Health, Belgrade, Serbia; 10 Faculty of Sport and Physical Education, University of Belgrade, Belgrade, Serbia; 11 School of Health and Caring Sciences, University of West Attica, Athens, Greece; 12 Department of Physiology, Federal University of São Paulo, São Paulo, Brazil; 13 Medbase St. Gallen Am Vadianplatz, Vadianstrasse 26, 9001 St. Gallen, Switzerland

**Keywords:** Environmental sciences, Environmental social sciences

## Abstract

The 50-km ultra-marathon is a popular race distance, slightly longer than the classic marathon distance. However, little is known about the country of affiliation and age of the fastest 50-km ultra-marathon runners and where the fastest races are typically held. Therefore, this study aimed to investigate a large dataset of race records for the 50-km distance race to identify the country of affiliation and the age of the fastest runners as well as the locations of the fastest races. A total of 1,398,845 50-km race records (men, n = 1,026,546; women, n = 372,299) were analyzed using both descriptive statistics and advanced regression techniques. This study revealed significant trends in the performance of 50-km ultra-marathoners. The fastest 50-km runners came from African countries, while the fastest races were found to occur in Europe and the Middle East. Runners from Ethiopia, Lesotho, Malawi, and Kenya were the fastest in this race distance. The fastest 50-km racecourses, providing ideal conditions for faster race times, are in Europe (Luxembourg, Belarus, and Lithuania) and the Middle East (Qatar and Jordan). Surprisingly, the fastest ultra-marathoners in the 50-km distance were found to fall into the age group of 20–24 years, challenging the conventional belief that peak ultra-marathon performance comes in older age groups. These findings contribute to a better understanding of the performance models in 50-km ultra-marathons and can serve as valuable insights for runners, coaches, and race organizers in optimizing training strategies and racecourse selection.

## Introduction

An ultra-marathon is any race that exceeds the traditional marathon distance of 42.195 km or lasts longer than 6 h^1,2^. These races can be distance-limited (such as 50 km or 100 km)^3^ or time-limited (such as 6 h or 12 h)^4^. As such, a 50-km run is considered the shortest distance-limited ultra-marathon. The 50 km distance is the most popular ultra-marathon race, offering a challenging but achievable step up from the traditional marathon distance^2^. There has been significant scientific interest in this specific ultra-marathon race format. Studies have explored a range of topics related to this type of race, including performance trends^5,6^, the age at which the best performances are achieved^5,7,8^, age-related performance decline^5,9^, the sex difference in performance^5,10–12^, the aspect of inflammatory processes^13^, nutritional aspects^14^, exercise-induced influences on the heart^15–18^ and the foot strike pattern^19,20^.

One aspect that has not been explored is the country of affiliation of the fastest 50-km ultra-marathoners. It is widely acknowledged that runners from East African countries are the fastest in the marathon distance^21^. However, little comparable information is available for ultra-marathon races, especially for shorter distances like the 50-km race. Previous studies have investigated the country of affiliation of top performers in longer ultra-marathons, such as the 100-km^22,23^ and 100-mile races^24^.

One study found that Japanese runners were the fastest in the 100-km ultra-marathons^23^, while another study reported that Russians were the fastest in the same distance race^22^. For 100-mile ultra-marathons, the fastest women were found to be from Sweden, Hungary, and Russia, while the fastest men originated from Brazil, Russia, and Lithuania^24^. The exceptional results of the Russian athletes during this time span could have been affected by doping and the use of illicit substances^25,26^.

Regarding the age group presenting better results in long-distance events, Rust et al.^27^ demonstrated that in a 100-km ultra-marathon, runners in the age group 18–24 years were slower than runners in the older age groups. Therefore, it seems that very young runners do not achieve the best results in long-distance races. However, it is interesting to know which age group has the best results in 50-km ultra-marathon.

The aim of this study was to identify the country of affiliation and age of the fastest 50-km ultra-marathon runners, as well as the locations of the fastest races. While the dominance of East African runners in traditional marathon events is well documented, similar insights for ultra-marathons, especially at the shorter 50-km distance, are noticeably absent. Previous studies have explored the origins of top performers in more extended ultra-marathons, such as the 100-km and 100-mile races. However, to the best of our knowledge, this is the first study to analyze the country of affiliation for elite athletes in the 50-km category.

Based on the findings of previous studies, we hypothesized that there would be a significant diversity in the nationalities of the leading runners in the 50-km ultra-marathon, with a specific expectation that Russian athletes would emerge the fastest. Additionally, we predict that runners aged 35 and older will achieve the fastest times.

## Methods

### Data set and data preparation

For this study, official race results from the official DUV website (https://statistik.d-u-v.org) were obtained. Each race record included the participants’ age, gender, country of affiliation, the event location and year, and the average race speed in km/h. Data processing entailed general clean-up (discarding duplicates and incomplete/erroneous records) and classifying records into 5-year age groups. The country variables (‘athlete country’ and ‘event country’) showed high cardinality and records from countries with samples smaller than 10 records were filtered out to reduce noise.

### Statistical analysis

Histograms of the number of records and the average race speed by age/age group were visualized, displaying approximate Gaussian distributions. The variables ‘athlete country’ (the athlete’s country of affiliation) and ‘event country’ (the country where the race took place) were used to rank the countries by average race speed by aggregating by the country columns and then sorting by average race speed. Records from countries with less than 10 records were removed from the set to reduce noise and ensure that the results were statistically representative.

The resulting dataset contained 1,398,845 race records from 549,154 unique runners from 122 countries, participating in 50-km races held in 86 countries worldwide between 1894 and 2022. The descriptive statistical data in the ranking tables includes the number of records and the mean, standard deviation (std), max, and min values of the race speed.

In addition to this, an XG Boost regression model was built, with the following variables used as predictors or inputs to the model:

- Athlete_gender_ID
- Age_group_ID
- Athlete_country_ID
- Event_country_ID

These variables are the encoded versions of the original variables (‘athlete gender’, ‘age group’, ‘athlete country’, and ‘event country’). Athlete_gender_ID was encoded as 0 = female and 1 = male. Age_group_ID was encoded as the lowest value included in the age group (“18–24” becomes 18, “25–29” becomes 25, etc.). The Athlete_country_ID and Event_country_ID variables were encoded as per the country’s position in the descriptive country ranking tables. The predicted variable, or model output, was ‘race speed’ (km/h). Two evaluation metrics, MAE and R^2^, were calculated to assess the model's accuracy and behavior, along with the model relative features importance and prediction distribution plots. Following some basic hyper-parameter tuning, the model was trained and tested with the full sample (in-sample testing).

To further qualify the results, an MLR (Multivariate Linear Regressor) model and four individual ULR (Univariate Linear Regressor) models—all based on the OLS (Ordinary Least Squares) method were made. The results were then compared to the XGBoost model results to quantify the statistical importance of the variables. All data processing and analysis were done using Python (http://www.python.org/) and a Google Colab notebook (https://colab.research.google.com/).

### Ethical approval

This study was approved by the Institutional Review Board of Kanton St. Gallen, Switzerland, with a waiver of the requirement for informed consent of the participants as the study involved the analysis of publicly available data (EKSG 01/06/2010). The study was conducted in accordance with recognized ethical standards according to the Declaration of Helsinki adopted in 1964 and revised in 2013.

## Results

**Figure 1:**
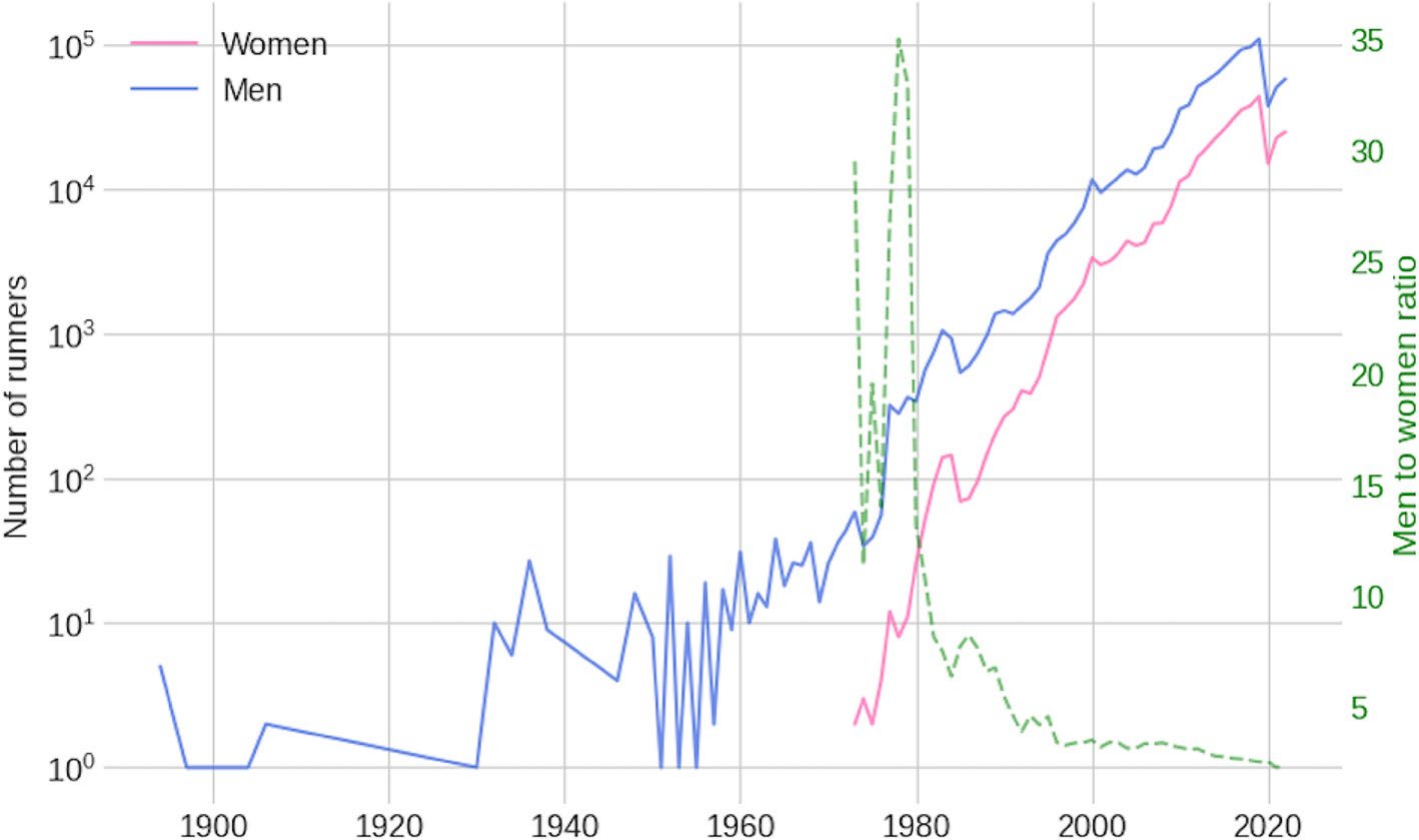
The number of runners and men-to-women ratio over the years.

After the dataset was processed and countries with less than 10 records were discarded, the dataset used to train and evaluate the model contained 1,398,845 race records (1,026,546 for men and 372,299 for women) from 549,154 unique runners from 122 countries, participating in races held in 86 different countries between 1894 and 2022. During this period, the overall number of women and men marathoners increased, and the men-to-women ratio decreased (Fig. 1).

### Runners’ country ranking

The dataset was grouped by the values in the ‘athlete country’ column and subsequently arranged in descending order based on the average (mean) race speed, with the fastest at the top. The resulting 122-country ranking is shown in Table 1, where the fastest runners originated from African countries (Ethiopia (ETH), Lesotho (LES), Malawi (MAW) and Kenya (KEN)).

**Table 1:** Athletes’ country sorted by average (mean) race speed.

	Athlete country	Race speed (km/h)					Country
		Count	Mean	Std	Min	Max	
0	ETH	46	14.087	2.785	5.415	18.505	Ethiopia
1	LES	379	13.053	3.128	5.185	18.248	Lesotho
2	MAW	104	12.346	2.233	6.487	17.769	Malawi
3	KEN	206	12.344	3.262	5.605	18.465	Kenya
4	MAR	103	11.718	3.897	3.586	17.523	Morocco
5	MOZ	45	11.414	2.052	3.777	14.027	Mozambique
6	ZIM	1117	11.377	3.066	4.834	18.009	Zimbabwe
7	BLR	446	11.346	2.374	3.972	16.826	Belarus
8	RWA	10	11.137	3.588	7.909	17.371	Rwanda
9	LTU	420	10.932	2.444	3.694	17.256	Lithuania
10	UKR	1226	10.759	2.291	3.524	17.678	Ukraine
11	LAT	611	10.334	2.441	3.697	16.602	Latvia
12	SWZ	234	10.223	2.046	4.004	16.579	Eswatini
13	ANG	31	10.167	1.839	5.938	12.822	Angola
14	CUB	16	10.135	2.163	5.016	12.913	Cuba
15	GER	61,559	10.115	1.950	2.206	17.741	Germany
16	JOR	80	9.935	2.482	4.079	16.428	Jordan
17	MNE	39	9.877	2.174	4.930	14.070	Montenegro
18	CMR	19	9.866	1.601	4.463	11.902	Cameroon
19	IRL	3296	9.843	2.475	2.606	17.729	Ireland
20	TUN	33	9.770	2.779	4.497	16.335	Tunisia
21	UGA	33	9.735	2.599	5.480	15.004	Uganda
22	FIN	1124	9.734	2.175	3.662	15.662	Finland
23	RSA	146,441	9.731	1.897	2.122	18.725	South Africa
24	HUN	4479	9.722	2.103	2.903	16.900	Hungary
25	CZE	4816	9.702	2.237	2.778	16.797	Czechia
26	SRB	377	9.685	2.850	3.209	16.180	Serbia
27	ZAM	107	9.549	1.777	5.437	15.997	Zambia
28	BOT	212	9.480	1.599	5.118	16.382	Botswana
29	NED	11,258	9.370	1.991	1.123	17.261	Netherlands
30	SVK	2156	9.367	2.766	3.258	16.524	Slovakia
31	ALB	62	9.340	1.702	5.910	12.757	Albania
32	NAM	425	9.305	1.222	5.034	14.198	Namibia
33	TAN	35	9.275	1.211	5.613	11.660	Tanzania
34	EST	160	9.249	1.991	3.507	13.589	Estonia
35	CRO	497	9.219	2.596	3.843	15.415	Croatia
36	COD	27	9.195	1.086	7.647	13.138	Democratic Republic of the Congo
37	MGL	114	9.118	3.044	4.351	16.071	Mongolia
38	RUS	7814	9.053	2.525	0.600	17.395	Russia
39	URU	258	8.997	2.720	3.363	15.214	Uruguay
40	SWE	12,083	8.942	1.985	2.402	16.868	Sweden
41	NOR	4722	8.906	2.292	3.067	17.736	Norway
42	ALG	58	8.873	2.182	3.837	13.873	Algeria
43	QAT	115	8.857	2.703	4.432	15.991	Qatar
44	BIH	120	8.841	3.248	3.244	15.780	Bosnia and Herzegovina
45	BEL	11,828	8.831	2.566	3.410	16.913	Belgium
46	EGY	33	8.776	2.787	4.548	14.302	Egypt
47	DEN	2862	8.706	2.214	3.140	16.805	Denmark
48	GUA	327	8.642	2.906	2.466	14.030	Guatemala
49	GRE	2844	8.600	2.178	3.541	15.570	Greece
50	ISL	133	8.555	2.196	4.463	14.589	Iceland
51	BOL	105	8.541	2.145	4.176	12.397	Bolivia
52	SMR	101	8.534	1.910	4.020	13.627	San Marino
53	ITA	46,182	8.519	2.226	2.083	17.415	Italy
54	IVB	14	8.510	1.084	6.197	10.520	British Virgin Islands
55	AUT	3939	8.464	2.267	3.333	15.893	Austria
56	NGR	42	8.386	1.665	5.854	15.991	Nigeria
57	GBR	32,330	8.303	2.252	2.206	17.847	United Kingdom
58	SUI	3534	8.277	2.389	3.064	16.837	Switzerland
59	LUX	257	8.228	2.002	3.627	13.137	Luxembourg
60	AUS	33,045	8.196	2.245	1.112	17.563	Australia
61	COL	417	8.181	2.770	3.898	14.949	Colombia
62	IRI	49	8.158	1.657	4.184	11.626	Iran
63	ESP	11,106	8.101	2.362	2.943	17.889	Spain
64	CAY	13	8.060	1.379	6.029	9.800	Cayman Islands
65	TPE	27,255	8.038	1.885	2.169	15.545	Taiwan
66	SLO	1601	7.924	2.186	3.584	15.509	Slovenia
67	CAN	42,202	7.893	1.882	2.802	17.799	Canada
68	FRA	64,875	7.834	2.114	2.589	17.921	France
69	ISR	936	7.817	2.148	3.319	15.278	Israel
70	POL	8076	7.763	2.202	3.219	17.329	Poland
71	BRA	6684	7.751	2.626	2.288	17.848	Brazil
72	USA	620,896	7.741	1.770	0.502	20.087	United States of America
73	PUR	47	7.714	1.943	3.838	11.719	Puerto Rico
74	NZL	4944	7.585	2.011	2.363	17.169	New Zealand
75	SYR	18	7.566	1.955	4.984	10.693	Syria
76	UAE	35	7.566	2.094	4.085	11.765	United Arab Emirates
77	IND	3807	7.482	1.891	2.501	15.708	India
78	KSA	14	7.435	2.643	5.048	15.841	Saudi Arabia
79	OMA	15	7.425	3.643	3.382	14.582	Oman
80	CYP	31	7.401	2.083	3.704	11.714	Cyprus
81	ESA	132	7.290	2.259	3.373	12.823	El Salvador
82	SRI	11	7.226	1.966	4.366	10.684	Sri Lanka
83	TUR	570	7.198	2.085	3.616	14.682	Turkey
84	JPN	41,157	7.181	2.207	2.803	17.498	Japan
85	ECU	709	7.120	2.653	3.472	14.048	Ecuador
86	CRC	924	7.106	1.531	3.063	12.252	Costa Rica
87	AND	49	7.104	2.890	3.855	14.930	Andorra
88	MLT	283	7.088	1.379	4.821	13.365	Malta
89	POR	5241	7.070	1.900	2.297	15.734	Portugal
90	PAN	19	7.056	1.479	5.226	10.375	Panama
91	MDA	80	7.016	2.696	3.546	12.377	Moldova
92	CAM	24	7.016	1.829	4.121	11.933	Cambodia
93	VEN	237	6.926	2.544	3.297	14.012	Venezuela
94	ARG	7708	6.837	2.120	2.774	16.087	Argentina
95	KOR	6515	6.824	2.111	2.392	15.654	South Korea
96	ROU	1080	6.813	3.004	2.597	17.396	Romania
97	MAD	13	6.805	1.803	4.109	10.938	Madagascar
98	LBN	20	6.750	2.284	4.119	12.267	Lebanon
99	MLI	12	6.737	1.084	5.433	8.042	Mali
100	NEP	253	6.692	2.058	2.758	14.151	Nepal
101	MEX	11,097	6.600	1.940	2.779	16.364	Mexico
102	PER	494	6.525	2.015	3.382	14.286	Peru
103	KGZ	12	6.483	2.638	3.552	11.134	Kyrgyzstan
104	CHI	1750	6.456	1.551	3.348	13.320	Chile
105	VIE	98	6.399	1.792	2.857	11.862	Vietnam
106	BUL	281	6.251	2.235	2.862	16.016	Bulgaria
107	HON	32	6.214	2.324	3.138	12.010	Honduras
108	PHI	9286	6.064	1.785	2.366	14.168	Philippines
109	DOM	67	5.944	1.278	4.188	10.346	Dominican Republic
110	SGP	2532	5.851	1.632	2.576	11.919	Singapore
111	MRI	360	5.792	1.489	3.683	11.826	Mauritius
112	KAZ	342	5.711	2.869	2.517	16.952	Kazakhstan
113	CHN	74,818	5.356	1.767	1.620	16.936	China
114	HKG	10,964	5.354	1.550	2.568	14.772	Hong Kong
115	MAS	9848	5.324	1.581	1.593	12.873	Malaysia
116	THA	11,600	5.093	1.163	2.757	13.685	Thailand
117	MAC	219	4.934	1.254	2.944	11.052	Macao
118	INA	877	4.872	1.383	2.780	12.613	Indonesia
119	PAR	216	4.867	1.549	1.678	11.345	Paraguay
120	BRU	688	4.778	1.435	2.112	10.059	Brunei
121	NCA	117	4.745	1.691	2.528	12.017	Nicaragua

Count (number of race records in each group), mean (average race speed of the race records in each group), std (standard deviation), min (minimum race speed in the group), max (maximum race speed in the group).

### Event country ranking

The dataset was aggregated based on the values in the ‘event country’ column, followed by sorting according to the average (mean) race speed, placing the fastest at the top. Table 2 displays the resulting ranking of 86 countries. The fastest race times were achieved in races held in Europe (Luxembourg (LUX), Belarus (BLR), and Lithuania (LTU)) and the Middle East (Qatar (QAT) and Jordan (JOR)).

**Table 2:** List of event countries sorted by average (mean) running speed.

	Event country	Race speed (km/h)					Country
		Count	Mean	Std	Min	Max	
0	LUX	15	11.360	0.892	9.839	12.698	Luxembourg
1	BLR	159	11.310	1.789	7.646	16.027	Belarus
2	LTU	234	11.211	1.849	5.587	17.256	Lithuania
3	QAT	363	11.160	3.017	4.636	17.427	Qatar
4	JOR	254	10.699	1.830	6.319	16.428	Jordan
5	LAT	374	10.602	2.414	5.313	16.602	Latvia
6	UKR	799	10.575	1.800	5.479	15.645	Ukraine
7	IRL	2449	10.441	2.347	3.710	17.925	Ireland
8	SRB	176	10.438	2.115	5.924	15.557	Serbia
9	GER	57,064	10.342	1.807	2.325	17.741	Germany
10	FIN	740	10.276	1.696	5.979	16.757	Finland
11	NED	11,721	10.216	2.001	1.123	17.678	Netherlands
12	CZE	5014	9.995	1.946	2.778	16.044	Czechia
13	SVK	2554	9.944	2.667	3.571	16.524	Slovakia
14	URU	97	9.855	1.763	5.695	15.214	Uruguay
15	EST	144	9.841	1.586	6.519	15.058	Estonia
16	RSA	154,825	9.768	1.893	2.122	18.725	South Africa
17	HUN	3792	9.754	1.902	2.903	16.900	Hungary
18	BOL	62	9.566	1.788	6.430	12.723	Bolivia
19	RUS	7542	9.078	2.422	0.600	17.395	Russia
20	SWE	12,206	8.981	1.957	2.402	16.868	Sweden
21	CRO	429	8.977	2.347	4.527	15.415	Croatia
22	NOR	4909	8.820	2.215	3.067	17.736	Norway
23	DEN	2380	8.696	2.079	4.608	17.114	Denmark
24	GRE	2792	8.685	2.213	4.091	15.570	Greece
25	CAY	19	8.616	1.658	6.029	11.595	Cayman Islands
26	PUR	28	8.542	2.308	5.714	12.672	Puerto Rico
27	ITA	46,393	8.540	2.230	2.083	17.523	Italy
28	MNE	101	8.500	3.532	3.209	14.943	Montenegro
29	ISL	94	8.499	2.146	4.581	13.557	Iceland
30	GUA	230	8.409	2.939	2.466	14.030	Guatemala
31	BEL	13,369	8.404	2.347	3.591	17.007	Belgium
32	GBR	28,253	8.349	2.187	3.391	17.847	United Kingdom
33	ESP	10,392	8.225	2.380	3.341	17.717	Spain
34	AUS	34,393	8.177	2.229	1.112	17.563	Australia
35	TPE	27,678	8.027	1.921	2.169	15.545	Taiwan
36	MGL	100	8.023	3.007	4.334	16.071	Mongolia
37	ISR	858	7.906	2.083	3.942	15.278	Israel
38	CAN	35,225	7.885	1.920	2.639	17.799	Canada
39	FRA	61,436	7.858	2.120	2.589	17.197	France
40	USA	631,046	7.751	1.769	0.502	20.087	United States of America
41	SUI	3495	7.748	2.391	3.040	16.238	Switzerland
42	BRA	5874	7.636	2.670	2.288	17.848	Brazil
43	AUT	5252	7.590	2.287	2.978	16.676	Austria
44	KSA	30	7.525	1.609	5.164	10.779	Saudi Arabia
45	POL	6891	7.493	1.869	3.219	17.189	Poland
46	IND	3480	7.433	1.781	2.558	14.904	India
47	CRC	821	7.367	1.292	4.565	12.252	Costa Rica
48	SLO	1496	7.364	1.839	3.584	14.127	Slovenia
49	NZL	4928	7.359	1.821	2.363	17.169	New Zealand
50	COL	264	7.354	2.288	3.898	12.540	Colombia
51	SGP	1833	7.179	1.386	4.378	14.578	Singapore
52	JPN	39,595	7.172	2.191	3.335	17.467	Japan
53	VIE	69	7.039	1.526	4.560	11.862	Vietnam
54	MLT	475	7.019	1.326	3.876	11.691	Malta
55	ARG	7396	6.878	2.143	2.774	16.087	Argentina
56	KOR	7058	6.842	2.250	2.392	15.835	South Korea
57	CYP	52	6.821	1.373	4.602	10.345	Cyprus
58	POR	4889	6.781	1.585	3.408	14.717	Portugal
59	TUR	452	6.764	1.610	3.852	13.346	Turkey
60	UAE	359	6.764	1.804	3.834	12.664	United Arab Emirates
61	MLI	18	6.550	0.926	5.433	8.042	Mali
62	PER	476	6.509	2.330	3.363	14.286	Peru
63	OMA	302	6.456	2.135	2.784	13.632	Oman
64	MEX	10,554	6.429	1.740	2.779	16.364	Mexico
65	CHI	2343	6.401	1.587	2.863	13.293	Chile
66	ECU	573	6.267	1.970	3.472	12.310	Ecuador
67	DOM	81	6.241	1.837	4.188	12.243	Dominican Republic
68	ROU	1697	6.177	3.347	2.206	17.889	Romania
69	BIH	80	6.167	1.591	4.154	13.199	Bosnia and Herzegovina
70	PHI	8819	5.985	1.759	2.366	14.168	Philippines
71	VEN	104	5.870	2.233	3.819	12.708	Venezuela
72	NEP	380	5.813	1.863	2.501	14.151	Nepal
73	MRI	534	5.625	1.228	3.683	9.720	Mauritius
74	BUL	167	5.565	1.121	3.361	8.743	Bulgaria
75	LES	468	5.532	1.149	3.686	9.291	Lesotho
76	BRU	267	5.469	1.099	4.196	10.550	Brunei
77	HKG	15,593	5.408	1.532	2.741	15.111	Hong Kong
78	CHN	75,181	5.386	1.810	1.620	16.936	China
79	MAS	10,788	5.263	1.599	1.593	13.064	Malaysia
80	AND	432	5.256	0.903	3.691	9.311	Andorra
81	THA	12,809	5.251	1.288	2.796	13.119	Thailand
82	PAR	245	4.635	1.210	1.678	11.322	Paraguay
83	INA	791	4.612	1.285	2.780	12.320	Indonesia
84	KAZ	360	4.585	1.078	2.517	8.283	Kazakhstan
85	NCA	365	4.551	1.170	2.528	9.434	Nicaragua

Count (number of race records in each group), mean (average race speed of the race records in each group), std (standard deviation), min (minimum race speed in the group), max (maximum race speed in the group).

### Model interpretability charts

**Figure 2:**
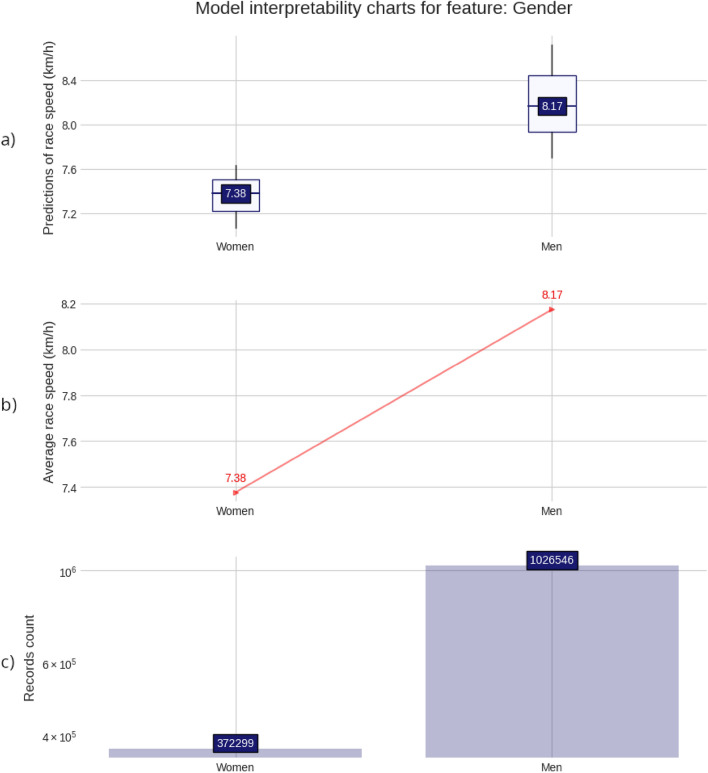
Model interpretability charts for feature: Gender. (**a**) Predictions of race speed (km/h); (**b**) Average race speed (km/h); (**c**) Records count.

**Figure 3:**
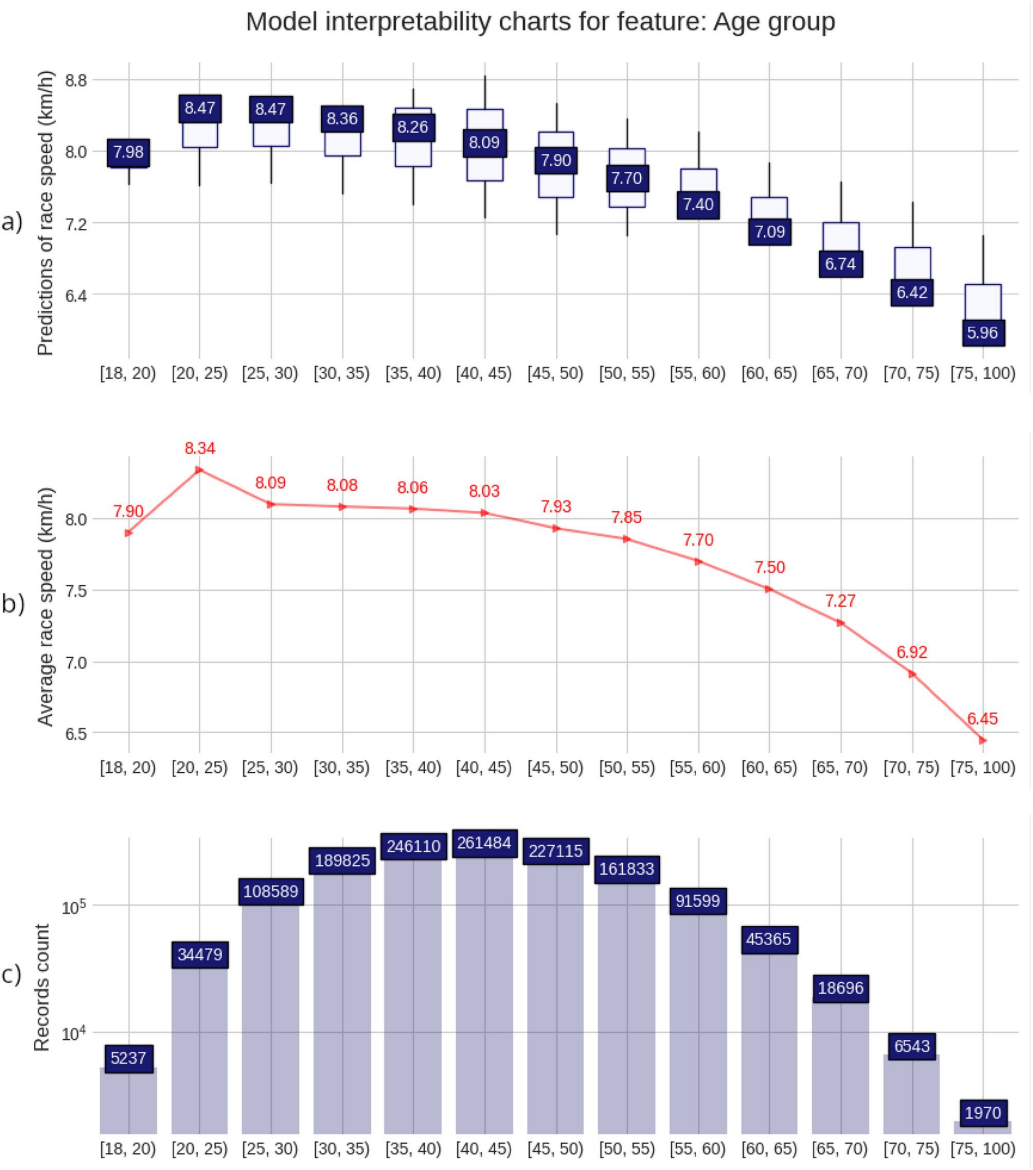
Model interpretability charts for feature: Age group. (**a**) Predictions of race speed (km/h); (**b**) Average race speed (km/h); (**c**) Records count.

**Figure 4:**
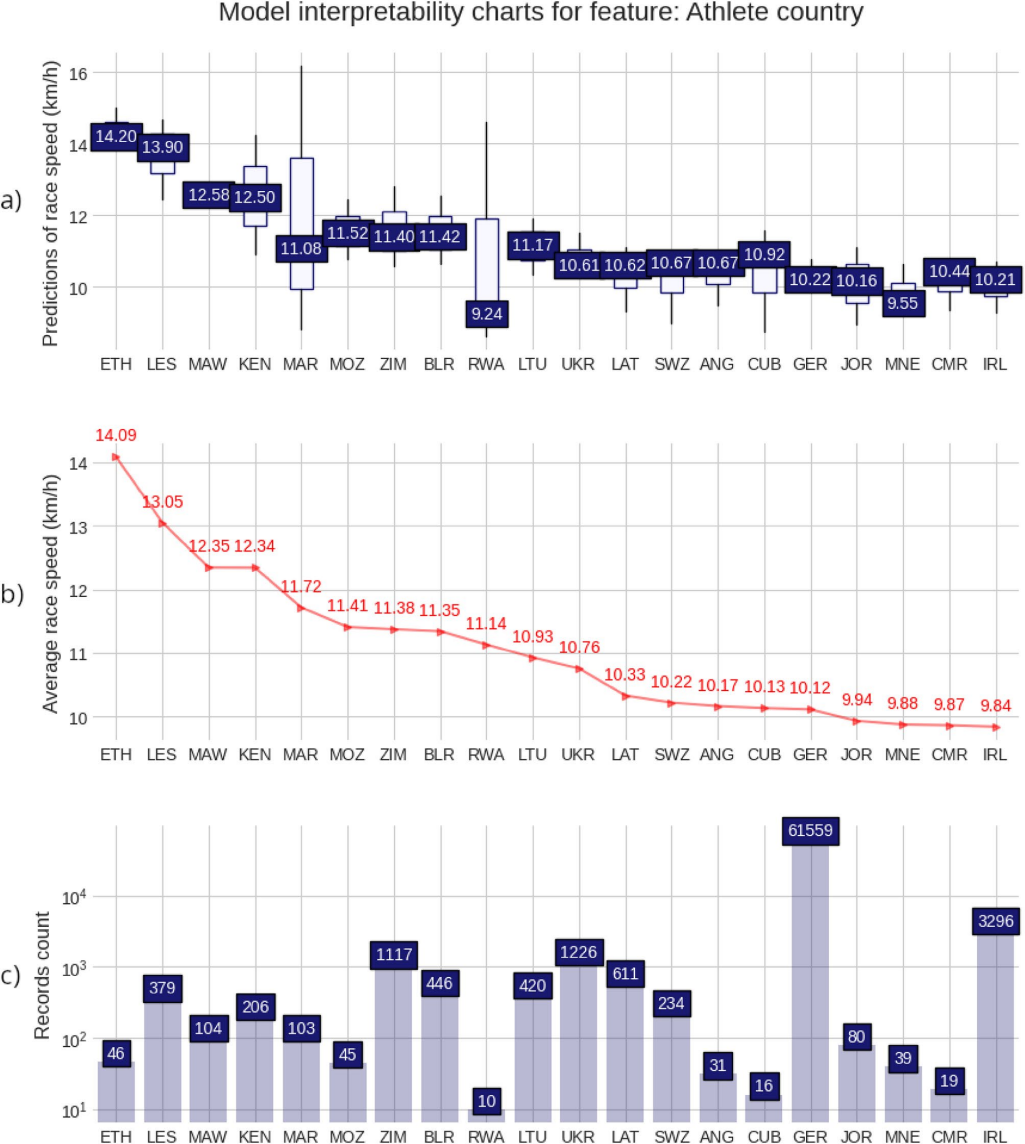
Model interpretability charts for feature: Athlete country. (**a**) Predictions of race speed (km/h); (**b**) Average race speed (km/h); (**c**) Records count.

**Figure 5:**
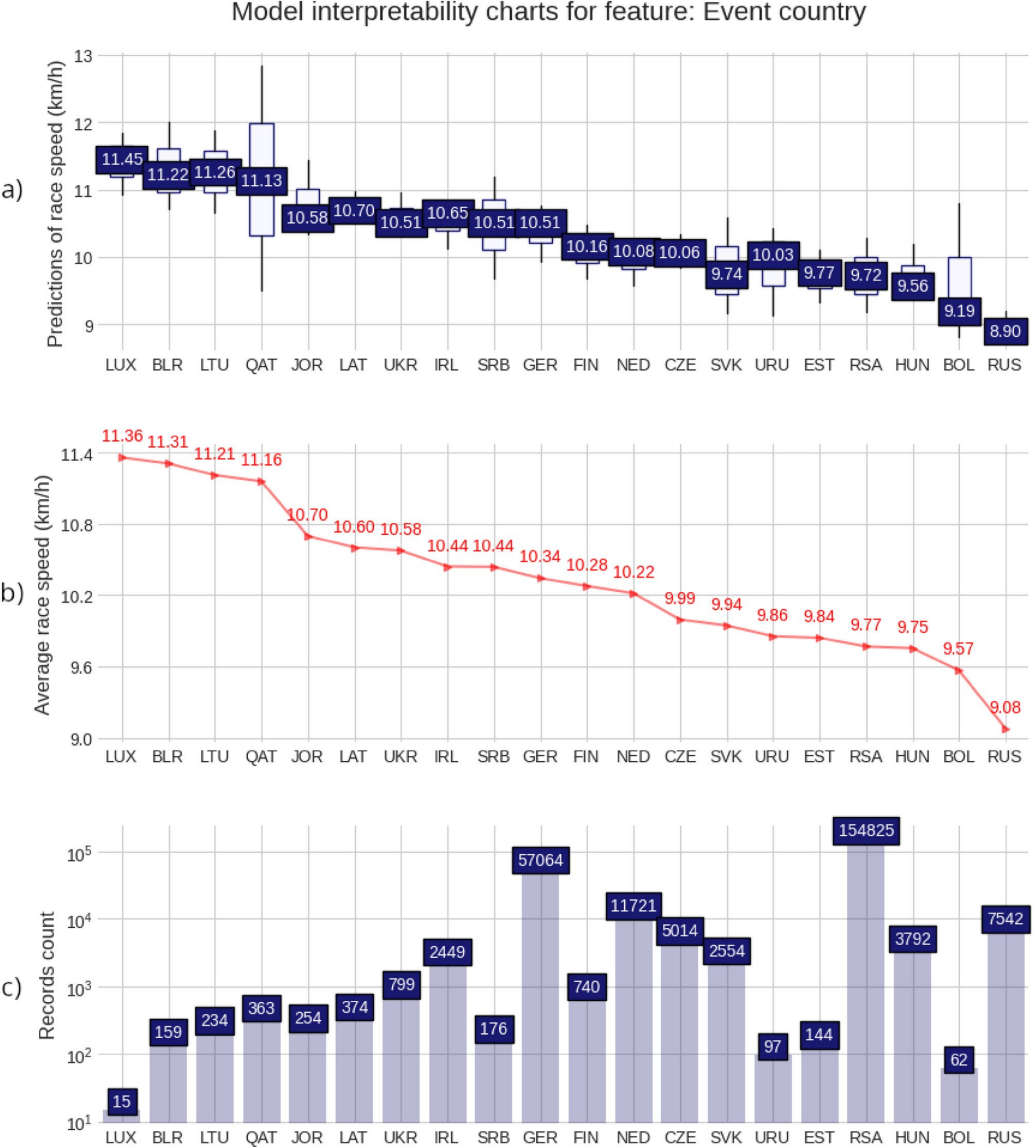
Model interpretability charts for feature: Event country. (**a**) Predictions of race speed (km/h); (**b**) Average race speed (km/h); (**c**) Records count.

The charts and plots presented in the Figs. 2, 3, 4, 5, provide a detailed visualization that combines a descriptive view of the full 50-km race sample with the predictive model insights. For each of the four predicting variables (age, gender, country of affiliation and country of event), a set composed of three charts is shown. A prediction distribution chart at the top as a boxplot chart with the 2nd quartile (median value) in the box label, a red line chart in the middle, representing the average race speed for each group, setting a target for the model prediction distributions, and a counting chart at the bottom showing the number of race records for each value of the predictor or group. For the ‘Age group’ and ‘Athlete gender’ predictors, all values were displayed. Still, for the ‘Athlete country’ and ‘Event country’ predictors, only the first 20 (the fastest 20) were displayed because of high cardinality (these match the top 20 countries in the ranking tables).

Men (8.17 km/h) were faster than women (7.38 km/h) (Fig. 2). Age group 20 (ages 20 to 24 years) was the fastest (8.34 km/h) in the 50-km races with running speed decreasing with age (Fig. 3).

The fastest runners’ countries were Ethiopia (ETH, 14.09 km/h), Lesotho (LES, 13.05 km/h), Malawi (MAW, 12.35 km/h), and Kenya (KEN, 12.34 km/h) as per the ‘athlete country’ ranking table (Fig. 4). The fastest 50-km races took place in Luxemburg (LUX, 11.36 km/h), Belarus (BLR, 11.31 km/h), Lithuania (LTU, 11.21 km/h), Qatar (QAT, 11.16 km/h), and Jordan (JOR, 10.70 km/h) as in the ‘event country’ ranking table (Fig. 5). The visualization further reinforced the earlier findings regarding the higher speed of runners from certain African countries and the optimal race conditions in specific European and Middle Eastern locations.

### Evaluation metrics and features importance

**Figure 6:**
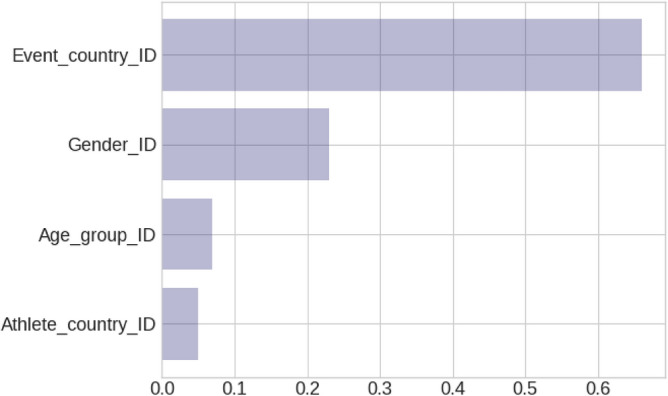
Evaluation metrics and features importance. Sample size 1,398,845. XGBoost trees 500. MAE (km/h 1.39). Feature importance Event_country_ID 0.66, Athlete_gender_ID 0.23, Age_group_ID 0.07, Athlete_country_ID 0.05.

The model for the 50-km race class exhibits an *R*^*2*^ = 0.36 coefficient of determination value, which indicates a weak but existing association of the predicting variables with the model output. In terms of feature importance, ‘Event country’ was the most important predictor (66%), followed by ‘Athlete gender’ (23%), ‘Age group’ (7%), and ‘Athlete country’ (5%) (Fig. 6). This hierarchy underscores the relative impact of these factors on race performance,

The MLR model achieved an *R*^2^ = 0.325, which is only marginally worse than our XGBoost results. All four predictors contribute statistically significantly to the MLR model output with a P value of 0.000 in all cases. The ULR models showed, although statistically significant, little statistical importance for ‘Athletes gender’ with an *R*^2^ = 0.025 and ‘Age group’ with an *R*^2^ = 0.006, while ‘Event country’ proved significant with *R*^2^ = 0.279 and Athlete country with *R*^2^ = 0.260 indicating that the ‘Athlete country’ and the ‘Event country’ variables are nearly equally important when used individually, suggesting a high correlation between them (e.g. runners in each country events were mostly affiliated to that same country).

## Discussion

The primary objective of this study was to investigate the country of affiliation of the fastest ultra-marathoners in the 50-km race category. Another aim was to identify the countries where the fastest 50-km ultra-marathon races are held and the age of the fastest runners participating. The main findings were (i) the fastest runners in the 50-km ultra-marathon originate from African countries (Ethiopia, Lesotho, Malawi, and Kenya), (ii) the countries with the fastest 50-km racecourses are in Europe (Luxembourg, Belarus, and Lithuania) and in the Middle East (Qatar and Jordan) and (iii) the age group 20–24 years showed the fastest 50-km ultra-marathon times. The results refute the authors' hypothesis since the fastest runners were from African countries, and the age group of the fastest 50-km ultra-marathon runners was younger than expected.

### Runners from Ethiopia, Lesotho, Malawi, and Kenya are the fastest 50-km ultra-marathoners

The first finding was that runners from Ethiopia, Lesotho, Malawi, and Kenya were the fastest 50-km ultra-marathoners. Several factors contribute to the prevalence of runners from East African countries in long-running events, such as marathons and ultra-marathons. These include a genetic predisposition, adherence to a traditional diet, living and training at high altitudes, and sociocultural background^28,29^. It is important to note that the country's infrastructure requires Ethiopians to daily walk or run with heavy school bags for long periods of time^30,31^.

For a significant period, there has been a prevailing suggestion that genetic background significantly influences sporting potential by determining the anthropometric, cardiovascular, and muscular characteristics contributing to adaptation during physical training^32^. This has suggested that runners from East African countries possess an inherent genetic advantage that predisposes them to superior athletic abilities^32^. Genetic studies conducted on elite African runners have not identified any unique genetic makeup; instead, they underscore the substantial genetic diversity among the general population and elite runners from East African countries^33^. Based on the available evidence, the subjects’ phenotype, shaped by various factors over time, exerts a greater influence on their success in long-distance running than their genotype^34^.

However, Kenyan runners have been found to exhibit a significantly higher activity of the enzyme hydroxylacyl-CoA dehydrogenase, which plays a crucial role in generating energy from lipids^35^. This suggests that Kenyan runners may have a more efficient ability to derive energy from lipid sources than some of their competitors^35^. Currently, there is no available information regarding enzymatic activity among elite Lesothan, Malawian or Ethiopian distance runners.

Larsen et al.^36^ examined the anthropometric characteristics of Kenyan distance runners, revealing that their legs were 5% longer compared to elite distance runners from Scandinavian countries. Additionally, the Kenyan runners had thinner and lighter calves, weighing 12% less when compared to runners from Scandinavian countries. Supporting these findings, Saltin et al.^37^ demonstrated that Kenyan distance runners exhibited greater metabolic efficiency, particularly at race-pace running speeds, compared to runners from Scandinavian countries. These observations suggest that the inherent ectomorphic somatotype of elite Kenyan runners may contribute to their success on the track and roads by enhancing their biomechanical and metabolic efficiency. However, it should be noted that there is a lack of evaluation regarding the biomechanical and metabolic efficiency of Lesothan and Ethiopian distance runners.

A study examining the dietary patterns of long-distance runners from Africa has revealed that they comply with most nutritional guidelines for endurance runners^38^. The traditional Ethiopian diet consists of 13% protein, 23% fat, and 64% carbohydrates^38^. The traditional Kenyan diet consists of 10% protein, 13% fat, and 77% carbohydrate^39^. The national dish of Lesotho is a fermented sorghum porridge^40^. Some staple foods include cornmeal porridge covered with a sauce consisting of vegetables^40^. The carbohydrate portion of the diet primarily consists of vegetables, fruits, rice, and unrefined sugar^40^. Malawi’s culinary culture revolves around integral ingredients such as sugar, corn, potatoes, sorghum, and fish, including the staple food Nsima made from ground corn^41^. People from African countries have consumed these low-fat, high-carbohydrate diets for centuries, and their composition is consistent with research-based recommendations for endurance runners^29^. While these diets seem beneficial for training and excelling in middle- and long-distance running competitions, they do not appear to possess unique differences compared to the training diets of runners from other continents^29^. As a result, other factors beyond food play a significant role in determining athletic superiority, as these diets are unlikely to provide a significant distinctive competitive advantage. It is important to highlight that the high-carbohydrate diet maintains muscle glycogen but has a negative effect on high-intensity exercise performance^42^. However, that may not be an issue considering lower intensities as long-distance running.

Certain factors, such as total hemoglobin mass, may be influenced by the environment where elite runners from the Kalenjin people in Kenya and the Arsi people in Ethiopia live and train^29^. The Kalenjin and Arsi people have a long history of residing at higher altitudes ranging from 2000 to 2500 m^29^. In particular, Ethiopian elite runners originate from high-altitude areas exceeding 4000 m, with approximately 80% of the population residing at or above 2000 m^43^. Malawi’s central plateaus, reaching 760 to 1370 m, cover approximately three-fourths of the entire land area^44^. Lesotho is the sole sovereign nation on Earth that exists entirely at an elevation surpassing 1000 m^45^. Consequently, its lowest point, reaching a remarkable altitude of 1400 m, is the world's highest among all countries. More than 80% of Lesotho’s landmass resides at elevations exceeding 1800 m^46^. The environmental context of living and training at higher altitudes could potentially contribute to developing specific physiological characteristics, including total hemoglobin mass^29^.

For many people of Ethiopia, Lesotho, Malawi and Kenya, running is a routine aspect of daily life, often utilized for transportation or as part of household chores, and children frequently start running at a young age as their main method of travel to school^43,47^. A theory suggests that long-distance runners may achieve a higher maximal oxygen uptake (${\dot{\text{V}}\text{O}}_{2}$ max) due to their early exposure to extensive walking and running^29^. Again, this could explain their exceptional endurance-running performance in later years.

African countries, most prominent in Ethiopia and Kenya, have a strong running tradition, and many experienced coaches and trainers work with young runners to develop their skills and talents^28,48,49^. These countries have a well-established infrastructure for running, with numerous running camps and facilities that support the development of elite runners^48–51^. It is important to remember that extraordinary athletic achievements among specific populations undoubtedly result from the successful combination of numerous factors.

Considering the example of a marathon race that has ~ 8 km shorter distance than 50-km-races, with the exception of a study^52^, there has been a consensus that Kenyans and Ethiopians were the fastest runners. The exception was the analysis of "World Athletics" fastest marathon runners from 1999 to 2015, which found that Latvians and Ethiopians were the fastest women and men, respectively^52^. On the other hand, a study of trends in the "New York City Marathon" from 2006 to 2016 as well as separate research of 50 years, showed that Kenyans and Ethiopians were the fastest^53^. This observation was confirmed in another popular American race, i.e., the Boston Marathon, analyzing data from 1972 to 2018 as well as from 1897 to 2017^54,55^. Moreover, these two East African nationalities were the fastest in the "World Marathon Majors" (Boston, Berlin, Chicago and New York) and the "Stockholm Marathon" from 2000 to 2014^56^, as and marathon races held in Switzerland from 1999 to 2014 confirmed this observation^57^. Thus, the findings of the present study in 50-km races agreed with those in marathon races, which might be explained by the high affinity of these two race distances. The models predictor showed the highest output values for African countries underlining the expected result. It might be assumed that similar physiological and psycho-social characteristics would play an important role in performance for these race distances.

The effect of the socioeconomic status of the participants should not be ignored. Due to the higher socioeconomic status and, in turn, a higher participation rate of athletes from Europe, Asia, or North America, compared to the participation rate of African countries, their sample will forcefully be more heterogeneous with a lower average speed, resulting in a comparable higher average speed of athletes from African countries. Further studies could attempt a stepwise analysis to gain more insight into the prediction strength of the athletes’ country affiliation itself.

### The fastest 50-km racecourses are in Europe (Luxembourg, Belarus, and Lithuania) and the Middle East (Qatar and Jordan)

Another significant finding was that the fastest mean race times were recorded in races held in Europe (Luxembourg, Belarus, and Lithuania) and in the Middle East (Qatar and Jordan). This result is highlighted by the highest output of the event country predictor.

Although Luxembourg has the fastest mean race times, it should be considered an outlier. The high mean race speed is due to the exceptionally high minimal race speed compared to the other countries. Based on the minimal race speeds, which elevate the mean race speed, we can assume that the participating runners were well above the average participant in other races. This effect should be considered a limitation of this study since a high distribution of lower-performing runners will skew the mean downward, as seen in the example of the United States of America, which has the highest average race speed but is downgraded by the high number of slower participants. Upon that, for precise measures, events with mean race speeds should not be considered in future analysis.

All the races mentioned above share a common characteristic—they are held on flat courses with minimal elevation changes. The racecourses in Belarus (indoor)^58^, Lithuania (road race)^59^, Qatar (road race, flat trail race)^60^, and Jordan (road race)^61^ are known for their flat terrain, which greatly contributes to achieving faster race times. These races offer smoother terrain and predictable conditions, allowing runners to maintain a steady pace without hindrance from inclines or steep descents. In addition, a study has demonstrated that flat terrain race results have been affected by the new advanced shoe technology^62^.

In contrast, trail running races are characterized by a sequence of off-road sections that involve uphill and downhill segments, resulting in significant physiological and mechanical changes^63,64^. Uphill sections involve prolonged and intense concentric muscle actions, while downhill sections require eccentric actions in the lower limb muscle–tendon unit^65^. These muscle actions and the duration of contractions differ from those in level road running, which primarily involve repetitive and continuous stretch–shortening cycles in the lower limb extensors^66^. In level road running, the upward and downward movements of the center of mass are generally balanced, along with the positive and negative external work within each step^67^. However, during incline running, the “bouncing” mechanism gradually diminishes as speed and slope increase^67^. On positive slopes, the step period decreases, and the body’s downward movement is reduced, while on negative slopes, the step period increases, and the upward movement decreases^67,68^. Steep changes in slope also lead to noticeable alterations in ground reaction forces, including a decrease in normal impact force peaks and parallel braking force peaks, accompanied by an increase in parallel propulsive force peaks^68^. Consequently, the repeated variations in slope and the associated mechanical responses in trail running races are likely to influence the manner of muscular contraction and metabolic demands^69^. To sum up, flat terrain plays a crucial role in achieving faster race times by providing more predictable conditions that enable runners to maintain a steady pace^70^. This allows runners to sustain their rhythm throughout the race and optimize their energy usage.

It is important to consider that the racecourse alone does not determine the entire outcome of the race. Again, factors such as runners’ preparation, training methods, nutrition, and individual capabilities also play significant roles. A combination of favorable racecourse characteristics and various other factors contributes to the overall faster race times observed in these countries. Although environmental factors like humidity and temperature can influence performance, this study did not include them in its analysis because of the unreliable and incomplete data for the analyzed events.

### The fastest 50-km ultra-marathoners are in the age group 20–24 years

An unexpected finding was that the fastest 50-km ultra-marathoners were in the age group 20–24 years. Typically, the age range when the fastest ultra-marathon race times are achieved is around 35 years or older^7^. The average age for first-time ultra-marathoners has remained unchanged in recent decades^71^. Individuals participating in an ultra-marathon were approximately 36 years old and had prior experience competing in shorter distances for approximately seven years^71^. The average age for first-time ultra-marathoners has remained unchanged in recent decades^71^. Several studies have analyzed the age of the best ultra-marathon performance^27,72–75^, revealing that peak performance is generally achieved at an older age compared to the best performance in half-marathons and marathons^76^. For marathon racing, the best race time is typically achieved around the age of 30^77,78^, in ultra-marathons, the age of best performance has generally been observed to be around 35 years or older^4,7,79,80^, with the age of peak ultra-marathon performance seemingly increasing as race distance increases. In particular, in 50-km ultra-marathon running, the best performance age is usually around 39–40 years^7^. It is easy to justify this considering that the peak performance is near 30 years old^81^ and decline after 40^82^.

Furthermore, this finding might be explained in terms of the variation of participation by age group. It was observed that a much smaller number of runners was in the age group 20–24 years compared to the older age groups. This difference in participation might indicate that this age group might be considered as a relatively more ‘selective’ than the older and more ‘massive’ age groups.

In summary, our finding that the fastest 50-km ultra-marathoners were in the age group 20–24 years is unexpected. The analysis of our model shows that the average race speed decreases after the PDP peak for age group at 20–24 years continuously until approximately − 1.75 km/h for the age group 75+. This contradicts the general belief that peak performance in ultra-marathons is achieved at an older age. This suggests that younger runners may have an advantage in this race distance, and further research is needed to understand the factors contributing to this age group’s success.

## Conclusion

In conclusion, this study provides valuable insights into the country of affiliation and performance of the fastest 50-km ultra-marathoners. Runners from Ethiopia, Lesotho, Malawi, and Kenya emerged as the top performers in this race format, benefiting from genetic predisposition, traditional diets, high-altitude living and training, and sociocultural background. The fastest mean race times, on the other hand, were observed in Europe (Luxembourg, Belarus, and Lithuania) and the Middle East (Qatar and Jordan), attributed to flat racecourses, well-developed infrastructures, and favorable conditions. A surprising finding was that the fastest ultra-marathoners in the 50-km distance were in the age group of 20–24 years, challenging the notion of peak performance in older age groups for ultra-marathons. Further research is needed to understand the underlying factors contributing to the success of younger runners in this specific race distance.

## Data Availability

We have included official race results from the official DUV website (https://statistik.d-u-v.org) for this study. The datasets used and/or analyzed during the current study are available from the corresponding author on reasonable request.
